# Flavonoids in Microheterogeneous Media, Relationship between Their Relative Location and Their Reactivity towards Singlet Oxygen

**DOI:** 10.1371/journal.pone.0129749

**Published:** 2015-06-22

**Authors:** Germán Günther, Eduardo Berríos, Nancy Pizarro, Karina Valdés, Guillermo Montero, Francisco Arriagada, Javier Morales

**Affiliations:** 1 Departamento de Química Orgánica y Fisicoquímica, Facultad de Ciencias Químicas y Farmacéuticas, Universidad de Chile, Santiago, Chile; 2 Departamento de Ciencias Químicas, Facultad de Ciencias Exactas, Universidad Andrés Bello, Santiago, Chile; 3 Departamento de Ciencias y Tecnología Farmacéuticas, Facultad de Ciencias Químicas y Farmacéuticas, Universidad de Chile, Santiago, Chile; Oregon State University, UNITED STATES

## Abstract

In this work, the relationship between the molecular structure of three flavonoids (kaempferol, quercetin and morin), their relative location in microheterogeneous media (liposomes and erythrocyte membranes) and their reactivity against singlet oxygen was studied. The changes observed in membrane fluidity induced by the presence of these flavonoids and the influence of their lipophilicity/hydrophilicity on the antioxidant activity in lipid membranes were evaluated by means of fluorescent probes such as Laurdan and diphenylhexatriene (DPH). The small differences observed for the value of generalized polarization of Laurdan (GP) curves in function of the concentration of flavonoids, indicate that these three compounds promote similar alterations in liposomes and erythrocyte membranes. In addition, these compounds do not produce changes in fluorescence anisotropy of DPH, discarding their location in deeper regions of the lipid bilayer. The determined chemical reactivity sequence is similar in all the studied media (kaempferol < quercetin < morin). Morin is approximately 10 times more reactive than quercetin and 20 to 30 times greater than kaempferol, depending on the medium.

## Introduction

For many years, flavonoids have been studied for their role in a broad variety of beneficial properties on human health [1–5]. Some of the pharmacological properties of flavonoids derive from their capacity to act as antioxidant in biological systems against free radicals and reactive oxygen species (ROS) such as singlet molecular oxygen, and its capacity to locate inside membranes [6–13]. The flavonoid total capacity to act as antioxidant and free radical scavenger depends on both, their location in the biological membranes and the substituents present in the molecule [14–17]. Additionally, membrane fluidity is an important property on which almost all essential cellular functions (cell growth, solute transport, signal transduction, membrane-associated enzymatic activities) are extremely dependent [18]. Any change promoted by the presence of different substrates on this property can interfere with the normal cell function, yielding for example pathological processes [19].

Among ROS, singlet oxygen, O_2_(^1^Δ_g_), an electronically excited state of molecular oxygen plays an important deleterious and/or beneficial role, when is present in biological structures [20–22].

In living aerobic organisms, the main route for singlet oxygen generation is photosensitization. In vitro, photosensitization is a simple and controllable method for O_2_(^1^Δ_g_) production, requiring only oxygen, light of an appropriate wavelength, and a photosensitizer capable of transferring its energy to the oxygen in its ground state, yielding its first singlet excited state [23, 24].

Matsuura et al. studied the photosensitized oxygenation of hydroxyflavone [25]. Chou et al. in a later work studied the photooxygenation of 3-hydroxyflavone in both, normal and tautomer state by direct time-resolved studies [26]. Borsarelli et al. studied the kinetic and mechanistic aspects of flavonoid stability in aqueous and methanolic solutions in the presence of superoxide radical and singlet oxygen. For these compounds, total reactivity scale is dominated by the physical quenching (k_q_) being the chemical reaction (k_R_) several orders of magnitude lower, reason why this process usually has been neglected [27].

García et al. reported the stability of flavonoids in the presence of reactive oxygen species (ROS) generated by photo-irradiation of riboflavin and eosin. Quercetin and rutin are the poorest singlet oxygen quenchers, mainly as physical quenchers, while morin is considered a “*sacrificial scavenger*”, because 80% of the singlet oxygen deactivation corresponds to degradation [28].

The reactivity of flavonoids with O_2_(^1^Δ_g_) has been studied only in homogeneous media [25–31], there are no reports in biological systems as membranes.

Cell membranes have a very complex architecture [32, 33]. For this reason, simpler synthetic structures such as vesicles and liposomes are often used to mimic lipid bilayers [34–36]. On the other hand, reconstituted erythrocyte membranes (ghost) have been widely used in many fields of study due to their interesting properties and simple preparation method. They have been widely employed as a typical model for cell membranes instead synthetic lipids. Erythrocyte ghost membranes represent a convenient model to study the antioxidant effect because they are especially vulnerable to damage by oxidant species [37–39]. Also, the particular erythrocyte membrane composition makes them valuable model system to study the effect of protein enclosure on reactivity. Singlet oxygen quenching is caused by specific aminoacids in proteins [40] for example Ogilby et al. demonstrated that singlet oxygen reaction rate constant with tryptophan depends on both, its the localization (direct molecular environment) and how accessible it is [41]. Chaudhuri and col. studied the interaction of flavonoids with red blood cell membrane lipids and proteins. Flavonoids are found to cause appreciable quenching of the tryptophan fluorescence of the membrane proteins [42]. Furthermore, many studies have investigated singlet oxygen generation and deactivation by molecules located in polymers and nanoparticles mainly for use in photodynamic therapy [43–45].

The different regions in lipid membranes (aqueous medium, interface and hydrocarbon chains) allow the interaction with a wide variety of substrates [46–48]. Thus, electrostatically charged species are bound to the interface, and hydrophobic substrates locate inside the bilayer. The extent and site of these interactions depend on the balance of electrostatic and hydrophobic interactions [49–51]. The protective effect of flavonoids against free radicals is frequently associated to the lipophilicity of these compounds [52–55].

**Fig 1:**
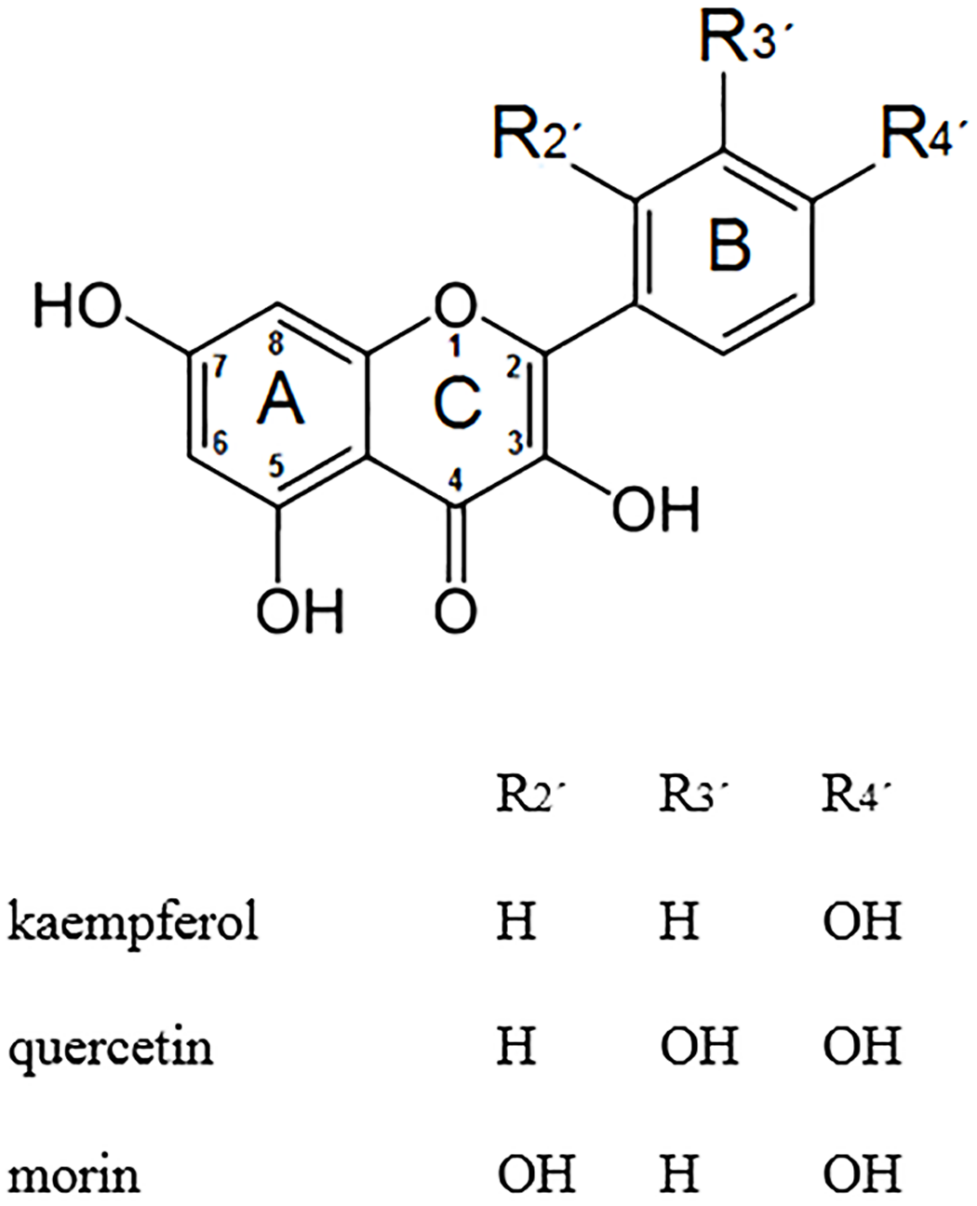
Chemical structures of flavonoids studied.

In this work, we studied three flavonoids (Fig 1: quercetin, morin and kaempferol) the relationship between their relative localization in liposomes (POPC, DPPC and mixtures) and in erythrocyte membranes, and their molecular structure with reactivity against singlet oxygen. In addition, changes in membrane fluidity induced by the presence of these flavonoids were evaluated.

## Materials and Methods

### Materials

Flavonoids (quercetin dihydrate, morin dihydrate and kaempferol hydrate) and lipids (1,2-Dipalmitoyl-sn-glycero-3-phosphocholine (DPPC) from Sigma and 1-Palmitoyl-2-oleoyl-sn-glycero-3-phosphocholine (POPC) from Avanti Polar Lipids (Alabaster, AL)) were used as received. Laurdan (6-dodecanoyl-2-(dimethylamine)-naphthalene) from Molecular Probes Invitrogen (Carlsbad, CA) and DPH (diphenylhexatriene) from Sigma were employed without further purification.

All solvents and reagents used were reagent grade, spectroscopic or HPLC quality. Water was purified and deionized using a Waters Milli-Q system.

#### Small unilamellar liposomes (SUVs) preparation

Small unilamellar liposomes (SUVs) were obtained by ultrasonication of lipids suspensions (1mM to 5mM) in pH 7.4 buffer phosphate, with a Cole Parmer Ultrasonic Homogenizer.

#### Large unilamellar liposomes (LUVs) and extruded liposomes preparation

Blank multilamellar large liposomes, MLVs, were prepared by the thin layer evaporation method. Lipids were dissolved in a small amount of chloroform. The solution was put in a small round-bottomed flask, the organic solvent was evaporated under nitrogen stream and the dry lipid films were maintained 2 hours under reduced pressure to remove solvent traces. Films were hydrated by adding an appropriate amount of 100 mM phosphate buffer pH 7.4, at 60°C, while shaking in vortex mixer. The phospholipid—buffer mixture was heated and shaken by short periods (four to six intervals of 1 minute), until homogeneous milky suspensions were obtained. Then, the homogeneous suspension was carefully frozen using a liquid nitrogen bath for 5 minutes and thawed in a water bath held at 60°C for the same period of time. This cycle was repeated five times. The MLVs suspensions were repeatedly extruded (10 times) through a polycarbonate filter (pore size 200 nm) using a 10 mL Lipex extruder (Northern Lipids Inc.).

#### Erythrocyte membranes preparation

Human freshly blood from healthy donor with sodium citrate was centrifuged at 12,000 rpm for 10 minutes at 4°C. Plasma and buffy coats were removed and the red blood cells (RBC) washed two times in pH 7.4 buffer phosphate. The pellet containing only red blood cells was then haemolyzed in hypotonic solution and centrifuged at 4°C. This cycle was repeated seven times. The hemoglobin free erythrocyte membranes obtained were dispersed in pH 7.4 isotonic buffer. Lipid concentration in erythrocyte membrane suspensions was calculated from phosphorous determination by spectrophotometric measurement of phosphovanadomolybdate complex [56,57] and the amount of total protein was determined by Brandford´s method [58].

This study was approved by the ethics committee of the University of Chile Faculty of Dentistry. The donor signed a written informed consent.

#### Flavonoid—liposomes / erythrocyte membranes solutions

Solutions of flavonoid incorporated to liposome or erythrocyte membranes were prepared by addition of small volumes of a standard stock solution of flavonoid in DMSO to liposome / erythrocyte membranes solutions. The mixture was homogenized in a vortex shaker and heated in a bath at 43°C for 30 minutes. Then the solution was slowly cooled up to 20°C and stored.

#### Size and zeta potential measurements

The average particle size and charge were analyzed using a zeta potential analyzer (Malvern Zetasizer Nano ZS90, Malvern, UK). The sample was diluted with pH 7.4 buffer phosphate prior to the determination of surface properties. Mean size and polydispersity index were obtained from 70 times measurements. Each sample was measured in triplicate and the mean value is represented.

#### Preparation of flavonoid loaded liposome and erythrocyte ghost membranes samples for HPLC analysis

Samples of flavonoid loaded liposomes and/or erythrocyte membranes for HPLC injections were prepared by diluting 400 μL of sample solution with 400 μL of ethanol in a conical plastic tube. The mixture was shaken in a vortex mixer for 2–3 minutes, left stand for 10 minutes and then centrifuged at 8,000 rpm during 30 minutes. Supernatant again centrifuged at 8,000 rpm during 30 minutes, it was finally separated and employed for HPLC injections.

#### High-performance liquid chromatography (HPLC)

The HPLC system was equipped with Agilent system 1100 and a photodiode array detector. HPLC analysis was performed using an Agilent ODS Hypersyl (5 mm-particle size, 20 cm × 4.6mm i.d.) column from Hewlett Packard. All experiments were carried out at 20°C of column temperature. The mobile phase consisted of a 49:1:50 v/v solution of water-phosphoric acid-acetonitrile with isocratic elution at a flow-rate of 1 mL/min. The diode array detector was operated at 358 nm with 4 nm of bandwidth. Injection volume was set at 100 μL.

#### Fluorescent probes incorporation

Laurdan and DPH were dissolved in ethanol and dimethyl sulfoxide, respectively. Liposomes and erythrocyte ghost membranes were incubated with fluorescent probes at a final concentration of 1μM, in a water bath held at 37°C for 30 minutes.

#### Fluorescence spectroscopy measurements

Steady state fluorescence measurements were made on a PC 1 photon counting steady state spectrofluorometer from ISS (Champaing, USA) at 25.0 ± 0.5°C and 37.0 ± 0.5°C. The fluorescence anisotropy of a fluorescent probe is defined as:

$${\text{R}}_{\text{ss}}=\;\frac{{\text{I}}_{\text{vv}}-{\text{ GI}}_{\text{vh}}}{{\text{I}}_{\text{vv}}+{\text{ GI}}_{\text{vh}}}$$ (1)

$$\text{G}=\;\frac{{\text{I}}_{\text{hv}}}{{\text{I}}_{\text{hh}}}$$ (2)

Where I_vv_ and I_vh_ are the fluorescence intensities emitted, respectively, parallel and perpendicular to the direction of the vertically polarized excitation light and G is the correction factor for the optical system given by the ratio of the vertically to the horizontally polarized emission components when the excitation light is polarized in the horizontal direction. In this work, the probe employed DPH was excited at 363 nm and the emission was measured at 426 nm [59,60].

The shape of Laurdan fluorescence spectra was evaluated from the fluorescence intensities measured at 490 nm and 440 nm by using the value of generalized polarization of Laurdan, GP, determined by using the expression of Parasassi et al. [61,62].

$$\text{GP}=\;\frac{{\text{I}}_{440\;}-{\text{ I}}_{490}\;}{{\text{I}}_{440}+{\text{ I}}_{490}}$$ (3)

### Determination of distribution and partition coefficients

To determine distribution coefficients of flavonoids in homogeneous medium, 2.5 mL of n-octanol and 2.5 mL of buffer (pH 1, 3, 5 and 7.4) were added in a flask and then stirred vigorously for 24 hours to allow system to reach equilibrium. The two phases were mutually saturated with one another. Then, in duplicate, an aliquot of ethanol solution of flavonoid (8 mM) was added to 2.5 mL of n-octanol saturated with buffer and/or buffer saturated with n-octanol. After mixing and stirring another 24 hours, the biphasic mixture was centrifuged at 2,000 rpm for 5 minutes at 25°C. The concentration of flavonoid in organic phase and the aqueous buffer phase were determined with HPLC analysis described above.

For the determination of distribution coefficient of flavonoids in heterogeneous medium, the samples were prepared by adding the appropriate amount of flavonoid solution in DMSO to the liposome suspension. Final sample volume, concentration of flavonoid and percentage of DMSO in the samples were 2.5 mL, 7x10^-5^M and 0.5%, respectively. Lipid concentrations varied from 0.25 mM to 2.5 mM. Prior to the measurements samples were incubated for 30 min at 37^°^C. Zero-order spectra of flavonoids in pH 7.4 buffer phosphate, liposomes and erythrocyte ghost membranes were obtained in the UV range 200–500 nm at a scan speed of 1200 nm min^-1^, data interval 1.0 nm and bandwidth 2 nm. To eliminate the residual background signal effects of dispersed medium as liposomes and erythrocyte membranes solutions, the derivative spectra, especially the second derivative, have been frequently used [63,64]. However, in this work, the best results were obtained using the first derivative spectra by instrumental electronic differentiation (Savitzky-Golay algorithm on Vision software). The changes produced in the first derivative spectra (^1^D) of flavonoid incorporated at different concentrations of phospholipids were registered (Eq 4).

$$\frac{\left[\text{L}\right]}{\Delta \text{D}}=\;\left(\frac{1}{\Delta {\text{D}}_{\text{max}}}\right)\left[\text{L}\right]+\;\left(\frac{\left[\text{W}\right]}{{\text{K}}_{\text{p}}\;\Delta {\text{D}}_{\text{max}}}\right)$$ (4)

Where,

[L]: molar lipid concentrations,

ΔD = ^1^D – ^1^D_0_, ^1^D represents the intensity of the first derivative of flavonoid solution in the presence of vesicles and ^1^D_0_ is the intensity of the first derivative of flavonoid in water or pH 7.4 buffer phosphate

ΔD_max_: is the extrapolated value of intensity of the first derivative when 100% of flavonoid is bound to the lipid

[W]: water concentration (55.5 mol L^-1^)

**Fig 2:**
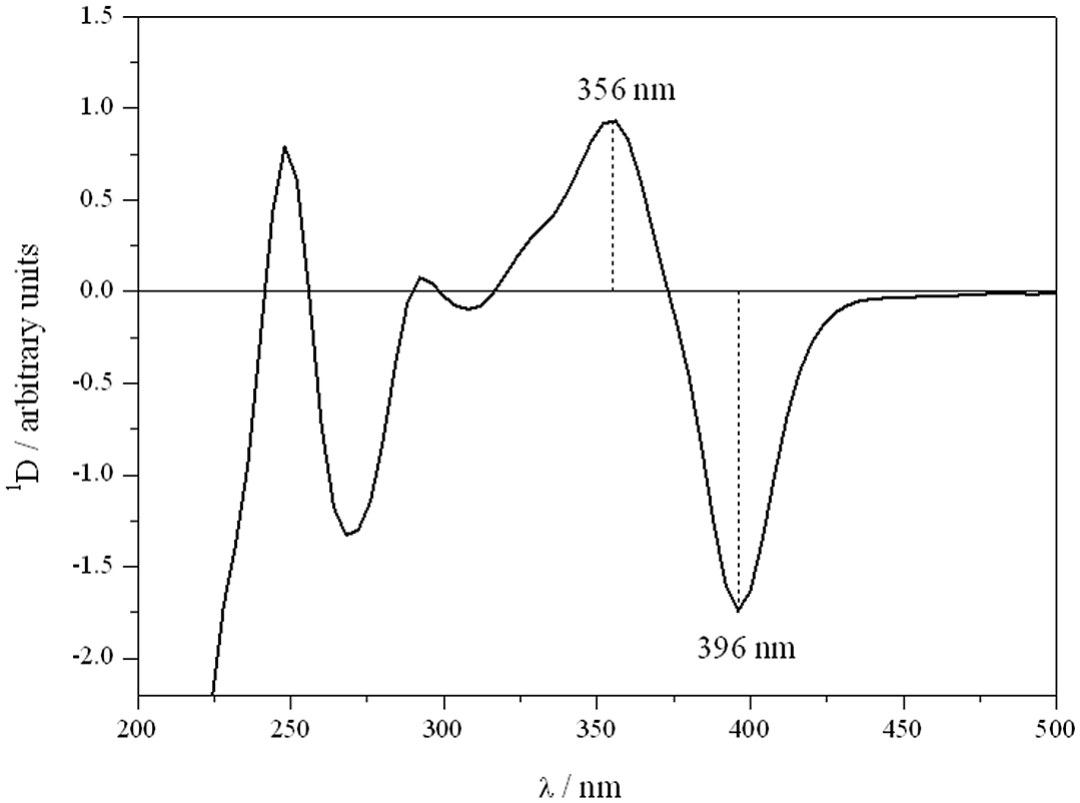
First derivative absorption spectrum of quercetin.

In the present work, we employed the absolute difference between the maximum and the minimum (^1^D_max-min_) expressed in arbitrary units, eg. for quercetin, the absolute difference between the maximum at 356 nm and the minimum at 396 nm (^1^D _356–396_) (Fig 2).

### Kinetic measurements

Experimental chemical reaction rate constants were determined in microheterogeous media using a 10 mL double wall cell, light-protected by black paint. A centered window allowed irradiation with light of a given wavelength by using Schott cut-off filters. Circulating water maintained cell temperature at 25 ± 0.5°C and 37 ± 0.5°C. The irradiation of the sensitizer was performed with a visible, 200 W, Par lamp. Flavonoid consumption was monitored by HPLC.

Time-resolved near-infrared phosphorescence measurements in erythrocyte ghost membranes were achieved by means of a PicoQuant FluoTime 300 fluorescence lifetime spectrometer. A PLS 575 LED-head was employed as the pulsed light source, in the burst mode. Luminescence of singlet oxygen was monitored at 1270 nm using a Hamamatsu NIR-PMT detector (H10330-45) and Fluofit software for the data analysis.

### DFT calculations

Kaempferol, quercetin and morin geometries were optimized at the DFT level employing the hybrid functional B3LYP [65–67] and 6–311++G** basis set for all atoms. Optimization were carried out in vacuum using tight convergence criteria and a fine grid for the numerical integration of the exchange-correlation contribution to B3LYP. Vibrational frequencies were calculated at the same level of theory in order to corroborate that the geometries correspond to an energy minimum. All calculations were performed using NWChem [68].

## Results and Discussion

### Characterization

The mean particle size of vesicles prepared by sonication ranges between 157 ± 5 nm (POPC /DPPC) and 163 ± 7 nm (DODAC). Polydispersity indices have values from 0.056 (DODAC) to 0.105 (POPC/DPPC), revealing high size homogeneity of these vesicles. Prepared erythrocyte membranes have an average size of 710 ± 23 nm which is much smaller than the average size of an intact human erythrocyte (8 μm) [69]. No significant changes in size and Zeta potential were observed upon addition of various concentrations of flavonoids (1 x 10^−5^ M to 1 x 10^−4^ M) to DODAC vesicles, POPC/DPPC liposomes or erythrocyte membranes, after incubating them for 30 minutes at 25°C and 37°C. The greatest change in Zeta potential is presented by morin in DODAC vesicles, modifying the surface charge from +73.1 mV up to +65.4 mV. In the absence of flavonoids the erythrocyte membranes Zeta potential is -13.5 mV and, for example, with the addition of 9.5x10^-5^ M of quercetin this value varies to -14.3 mV. For erythrocyte membranes Zeta potentials of -10 mV to -14 mV have been reported in previous studies [70].

### Partition and distribution

The distribution of morin, quercetin and kaempferol in hydrophobic / hydrophilic systems (n-octanol / buffered medium; lipid membranes / external aqueous phase) are shown in Table 1.

**Table 1:** Distribution (log D) of flavonoids in n-octanol, DODAC vesicles, POPC/DPPC.

	log D (distribution)			
Flavonoid	octanol / pH 7.4 buffer	DODAC vesicles in water	POPC/DPPC liposomes (7:3) in buffer pH 7.4	Erythrocyte membranes
kaempferol	1.25 ± 0.02	5.24 ± 0.15	4.55 ± 0.09	4.21 ± 0.29
quercetin	0.76 ± 0.09	4.61 ± 0.13	4.22 ± 0.04	4.03 ± 0.17
morin	0.42 ± 0.03	4.22 ± 0.09	3.87 ± 0.08	3.54 ± 0.28

Log D values show that quercetin and kaempferol have similar hydrophobicity, due to their structural similarities. However, morin, sharing these similarities in structure, has a lower value. Differences observed for quercetin and morin, could be due to the OH group increased interaction of n-octanol with the neighboring hydroxyl in the B ring of quercetin, which results in increased solubility of this flavonoid in n-octanol. Rothwell et al. reported that the log P value of quercetin (log P = 1.82) was significantly lower than kaempferol (log P = 3.11), i.e. quercetin is more hydrophilic than kaempferol. It is probable that the significantly higher log P (3.11) correlates with the absence of the catechol moiety in kaempferol, favouring partition to the n-octanol phase [71]. Oteiza et al. studied the distribution of 24 phenolic structures, reporting the following order of lipophilicity: myricetin > kaempferol ≥ quercetin > morin > catechin >epicatechin [47]. These results are consistent with the order of hydrophobicity of flavonoids obtained in this work.

For many years, n-octanol-water partition coefficient has been employed as a tool in studies of structure-activity and has been an useful indicator of hydrophobicity of molecules in hydrophilic-lipophilic systems [72,73]. Currently, several reports in the literature discuss if an apolar organic solvent-water partition coefficient can be employed as a representative scheme for the situation of a compound in biological medium. Seydel reported discrepancies between the values obtained for drug partition in an n-octanol / water system with respect to the distribution in lipid membranes [73]. Partition with n-octanol presents some disadvantages; among them, this system is not able to mimic the pronounced interfacial character given by the microenvironment formed by water and polar groups from the membrane phospholipids, and the presence of a strong ionic interaction of charged molecules with the polar head of these lipids [73].

The distribution of flavonoids is highly dependent on the position of hydroxyl groups. The ionized fraction of these compounds depends on the hydroxyl groups pKa and the medium pH. According literature in a buffered medium at pH 7.4, only the hydroxyl located at position 2´ of B ring from morin would be ionized [74]. At physiological pH, this hydroxyl is ionized, favoring its interaction with the surface of positively charged vesicles (DODAC). POPC / DPPC liposomes and erythrocyte membranes possess a potential close to neutrality, where the membrane—flavonoid interaction would be modulated by the effect of the electron density of the hydroxyl groups not ionized at this pH.

The relationship between partition or distribution coefficient and retention factor obtained by HPLC has been frequently used as a hydrophobicity predictor [75, 76]. Retention factor is defined as (t_R_—t_0_)/t_0_, where t_R_ is retention time of the studied compound (minutes) and t_0_ is void time (minutes). Retention factor between 0.5 and 20.0 is desired to properly resolve the first peak from void time and to avoid higher retention time for the last peak. In this work, the optimal chromatographic determination was obtained using a photodiode array detector set at 360 nm, corresponding to the flavonoids maximum absorption wavelength. Under these conditions, retention times for morin, quercetin and kaempferol were 7.3, 9.6, and 16.1 minutes, respectively and void time was 1.5 minutes for the method. Retention factors of 3.87, 5.40 and 9.73 (with solvent front as unretained compound) were found for morin, quercetin and kaempferol, respectively, indicating a very good separation of these compounds.

**Fig 3:**
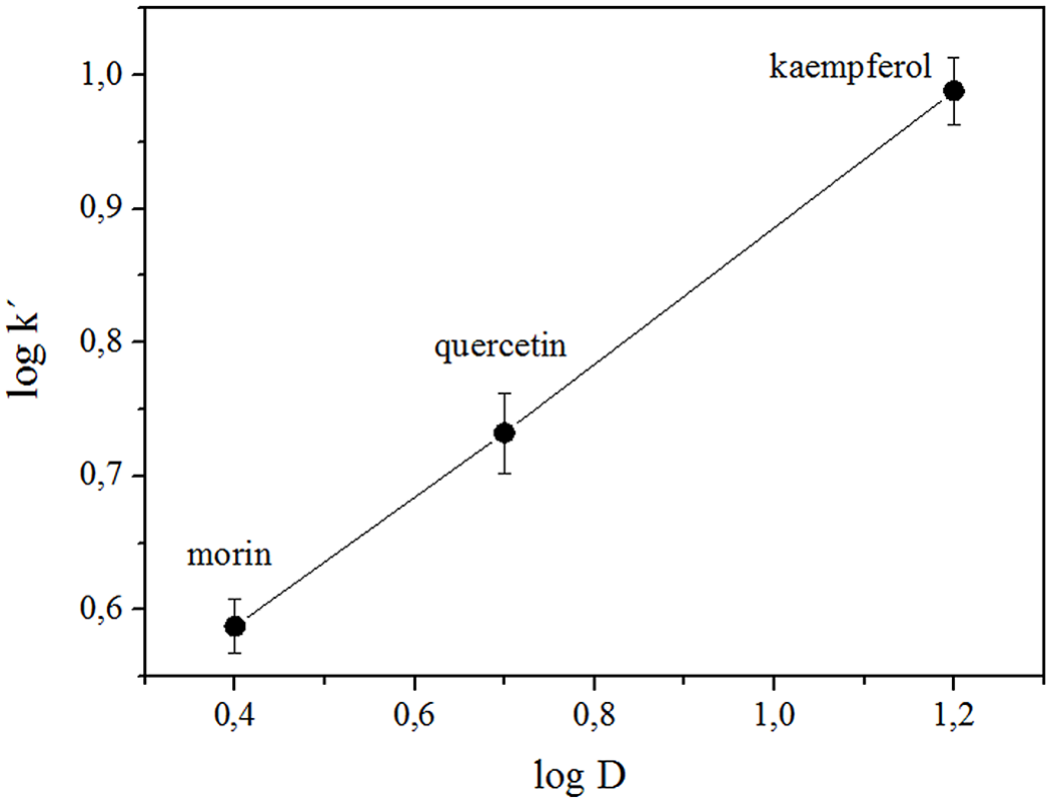
Plot log k´vs log D (n-octanol/ pH 7.4 buffer) of the flavonoids studied.

The good correlation obtained for the plot of log k´ vs log D (n-octanol/ pH 7.4 buffer) (Fig 3) for the studied flavonoids confirms that k’ as a good hydrophobicity predictor for these systems.

### Relative location in micro-heterogeneous media

In this work we studied the relative location of flavonoids in the micro-heterogeneous medium, using fluorescent probes (Laurdan and DPH). Laurdan is found in very low concentration in water, and all of its emission will come from the membrane. For membranes in gel phase state, its emission is centered at 440 nm, while in fluid phases emission maximum is located at 490 nm [61,62].

**Fig 4:**
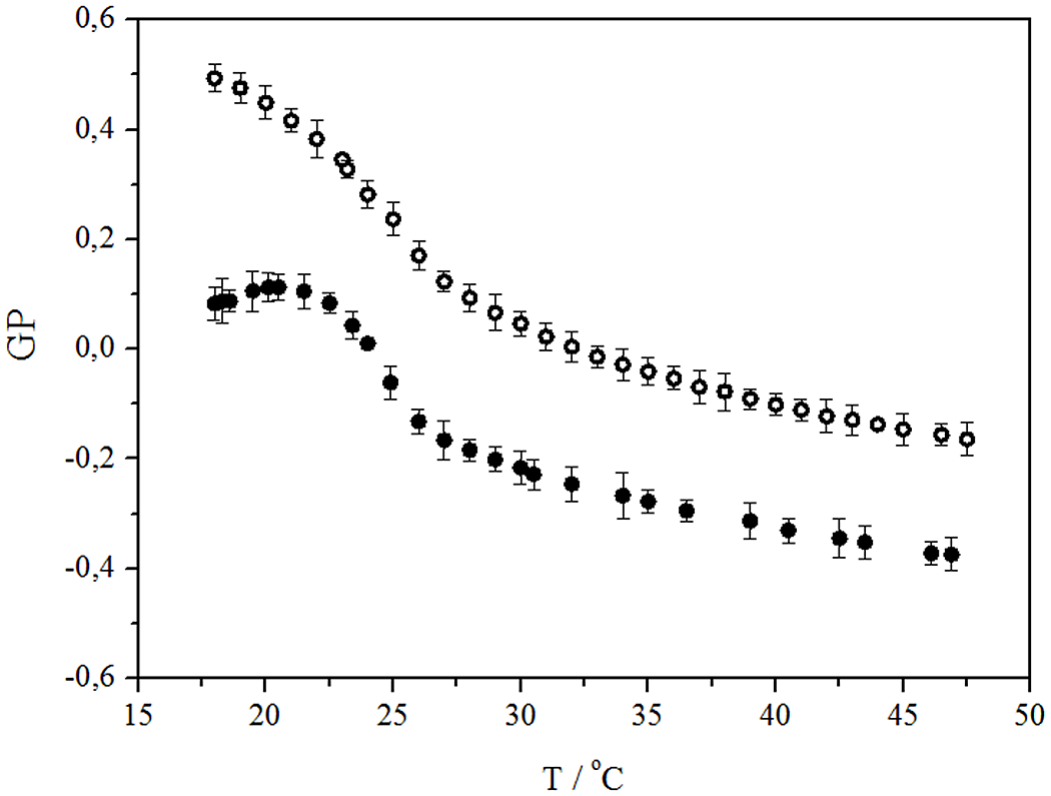
Laurdan generalized polarization (GP) as a function of temperature. POPC / DPPC liposomes (o) and POPC / DPPC liposomes with addition of 9 x 10^−5^ M of morin (•).

Membranes formed by POPC are more fluid than those made of DPPC because an unsaturation in one of its hydrocarbon chains, mimicking better what would happen in a biological system (erythrocytes membrane). The results show that GP values are independent of the liposome size, so the curvature radius does not significantly affect nor membrane structure and probably neither its fluidity. The fluorescence study showed that in the temperature range below the phase transition (Tm) of POPC / DPPC liposomes the GP initial value is high (+0.5), and gradually decreases to a value of -0.1 at 48°C. This indicates a greater possibility of water access probably due to greater thermal agitation of the surfactant molecules (Fig 4). Below T_m_, The addition of flavonoid (9.5x10^-5^ M) produces a large decrease in the GP value (+0.1); therefore its presence fluidizes the membrane, which indicates that the flavonoid shares the same location with Laurdan. For saturated phospholipids typical GP values around +0.5 − +0.6 have been reported for the pure gel phase, and for the pure crystal liquid state values range between -0.3 and -0.4.

**Fig 5:**
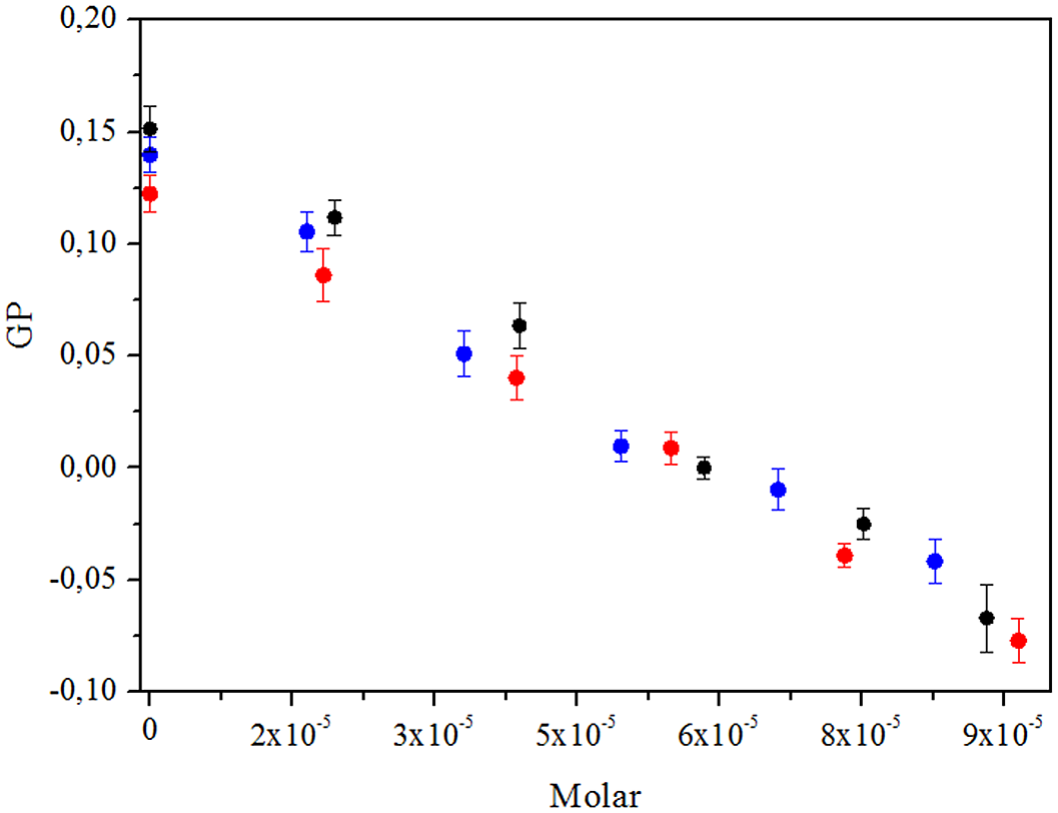
GP curves in function of the concentration of flavonoid incorporated in POPC / POPC liposomes. morin (•), quercetin (blue dot) and kaempferol(red dot).

Fig 5 shows Laurdan GP as a function of flavonoid concentration. The small difference among curves indicates that these three compounds promote similar alterations in liposomes and erythrocyte membranes. In the absence of flavonoids, the GP value in erythrocyte membrane was 0.47 and 0.32 at 25°C and 37°C, respectively, values that decreased to +0.17 and +0.11 when incorporating 7x10^-5^ M of flavonoid.

Moreover, increasing concentrations of each of the flavonoids do not produce noticeable changes in DPH fluorescence anisotropy, discarding the location of these antioxidants in deeper regions of the lipid bilayer.

### Reactivity of flavonoids with singlet oxygen in microheterogeneous media

The reaction of singlet oxygen with a flavonoid, FLAV, involves physical quenching (deactivation) and/or chemical (reactivity) processes. The chemical reaction (k_R_) can be followed by monitoring the FLAV consumption in time. In these experiments, the reaction kinetics fits to a pseudo first order process, according Eq 5:

Rate= kR [O2(Δ1g)][FLAV]= kOBS[FLAV] (5)

Where k_R_ is the chemical rate constant of singlet oxygen with the flavonoid, [O_2_(^1^Δ_g_)] is the singlet oxygen steady state concentration and k_OBS_ is the observed or experimental pseudo first-rate constant obtained from steady-state experiments. Under strict control of experimental conditions such as concentration of photosensitizer, light intensity, temperature and geometry of the cell, the generated singlet oxygen concentration remains constant. In this way, the ratio between the k_OBS_ (1 and 2) allows to compare the chemical reactivity.

$$\frac{{\text{k}}_{\text{OBS},1}}{{\text{k}}_{\text{OBS},2}}=\;\frac{{\text{k}}_{\text{R},1}}{{\text{k}}_{\text{R},2}}$$ (6)

Table 2 shows the values obtained for the experimental rate constants in different micro- heterogeneous media. The order of reactivity obtained is similar in all micro-heterogeneous media studied (kaempferol < quercetin < morin). No significant changes were seen in the reactivity depending on the phospholipid chains flexibility (POPC) or in the presence of proteins (erythrocyte membranes).

**Table 2:** Experimental rate constants (k_EXP_) results at different microheterogeneous media.

	Experimental rate constant (k_EXP_) / seconds^-1^			
Flavonoid	POPC liposomes	POPC/DPPC (7:3) liposomes	DPPC liposomes	Erythrocyte membranes
kaempferol	(1.32 ± 0.06) x 10^−4^	(1.74 ± 0.08) x 10^−4^	(2.75 ± 0.01) x 10^−4^	(1.61 ± 0.17) x 10^−4^
quercetin	(4.20 ± 0.13) x 10^−4^	(3.60 ± 0.09) x 10^−4^	(5.11 ± 0.50) x 10^−4^	(5.32 ± 0.78) x 10^−4^
Morin	(38.3 ± 1.1) x 10^−4^	(36.5 ± 1.5) x 10^−4^	(57.1 ± 0.32) x 10^−4^	(46.0 ± 3.5) x 10^−4^

The ratios between the experimental rate constants show that morin reactivity is almost 20 to 30 times higher than kaempferol and 10 times greater than quercetin (Table 3). The same trend was observed in homogeneous medium (ethanol) where total rate constants of morin, quercetin and kaempferol are 65.7 x 10^5^ M^-1^s^-1^, 5.7 x 10^5^ M^-1^s^-1^ and 2.8 x 10^5^ M^-1^s^-1^, respectively, with morin / kaempferol ratio of 23.5 and morin / quercetin of 11.5 [31].

**Table 3:** Ratio of experimental rate constants (k_EXP_) at different microheterogeneous media.

	Ratio between experimental rate constants			
Flavonoids	POPC liposomes	POPC/DPPC liposomes (7:3)	DPPC liposomes	Erythrocyte membranes
quercetin / kaempferol	3.2	2.1	1.9	3.3
morin / kaempferol	29.0	20.1	20.7	28.6
morin / quercetin	9.1	10.1	11.2	8.6

Among the studied compounds, morin has the highest reactivity. Morin is structurally similar to quercetin, differing only in the position of a hydroxyl group in the B ring.

Neglecting ionization of the hydroxyl groups or differences in electron delocalization dependent on the planarity of the molecule, this increased reactivity may be possibly explained by the direct activation of the double bond in position C2-C3 of C ring. In B-ring of quercetin and morin, the hydroxyl electrons in R4´ would be delocalized in the conjugated π system, producing activation of ortho and para positions, corresponding to C-3´, C-5´ and C-1´ positions, respectively. Delocalized electrons from the OH in R4´ would increase the electron density of double bond in position 2–3 from the C ring, which also receives the direct influence of the hydroxyl in R3 from the same ring.

However, at physiological pH, ionization of polyphenols is an important factor than must be heed. The flavonoids studied have four or five ionizable OH groups and obviously speciation is dependent on their pKa´s. Different pKa values for these flavonoids have been reported, which depend on the method used for their determination [74–78].

Rafols et al. stated that flavonols exhibit conjugation between the ring A and ring B, which will facilitate the deprotonation [78]. Musialik et al. stablished a structure-activity relationship indicating that in polar solvents the flavonoids react much faster with free radicals because the OH group at position 7 in ring A is deprotonated. In general, the pKa of 7-OH group is similar for all flavonoids (7.5 to 8.5), and the presence of other hydroxyl in positions 3, 5, and 6 does not considerably alter the acidity of 7-OH group. However, morin has a pKa of 5.2 for 2´-OH group being more acidic than quercetin and kaempferol. In order to explain this strong acidity these authors suggest the existence of a tautomeric form involving a hydrogen bond with the hydroxyl at position 3, stabilizing this deprotonated form observed. [74].

The study of the influence of hydroxyl deprotonation on the photo-oxidation of phenols must be focused on the balance between physical and chemical quenching contributions at each pH. The reaction rate constant for each ArO- species are between one and two orders of magnitude higher than that for the undissociated species [79].

The value of k_T_ is strongly dependent on the structural properties of the phenol, on the solvent, and on the phenol ionization degree. For example, k_T_ for α-tocopherol shows considerable increase with solvent polarity [80].

**Fig 6:**
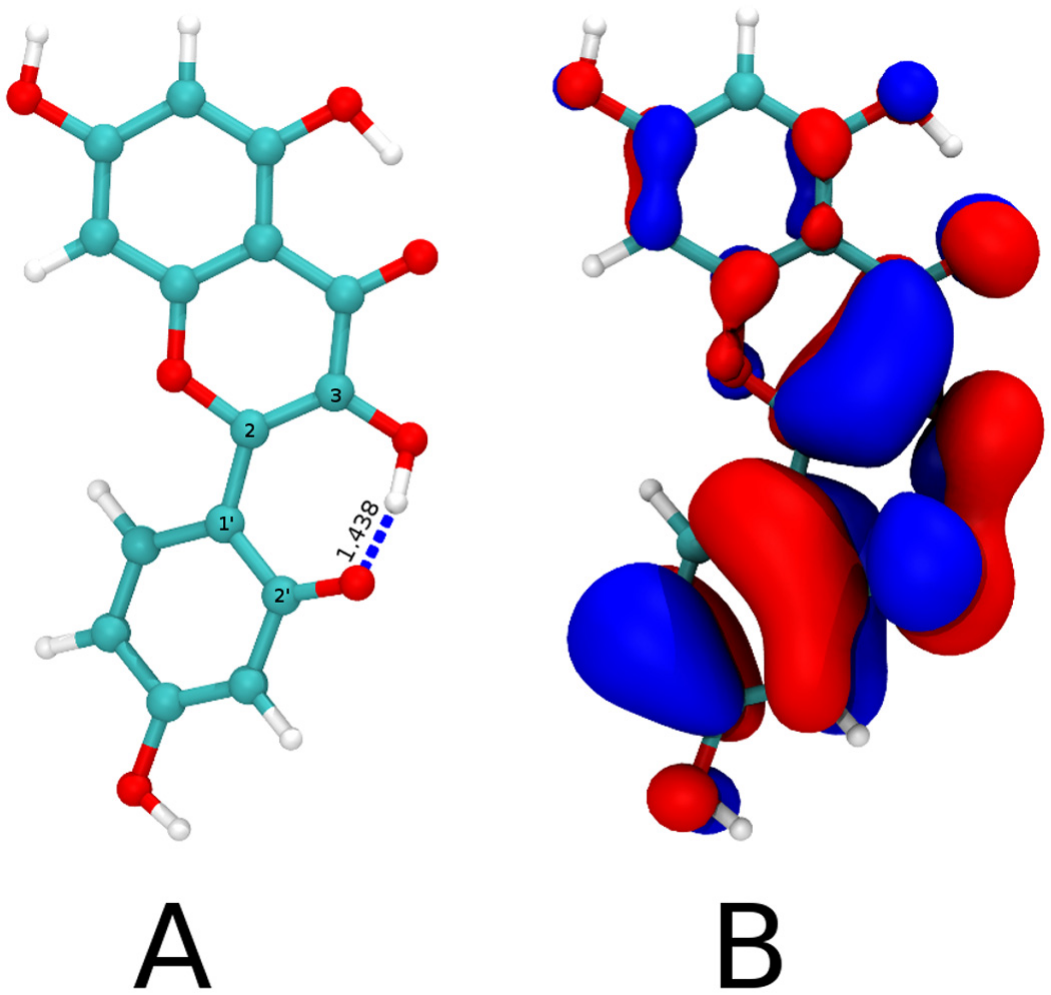
Morin deprotonated. Panel A shows the optimized geometry for morin deprotonated at 2’-OH at the DFT B3LYP/6-311++G** level with a dihedral angle C3-C2-C1’-C2’ equal to 27.6° and highlights the hydrogen bond between 3OH and 2’-O^-^ (1.438 Å, blue dashed line). White, cyan and red represent Hydrogen, Carbon and Oxygen atoms respectively. Panel B depicts the HOMO for the geometry in panel A (isovalue 0.02 a.u) showing large and localized electron density over the enol.

Chemical deactivation of singlet oxygen by flavonols is known to involve carbon double bond C2-C3 in ring C (Fig 1) following a [2+2] cycloaddition mechanism [26]. Also, it has been reported that photooxidation reaction rate of enolic tautomers of dicarbonyl compounds is greatly enhanced by the presence of fluoride ion or tetrabutilammonium hydroxide [81]. This effect has been attributed to hydrogen bond formation between the fluoride ion and the enol hydrogen which enhances its nucleophilicity [82]. Fig 6A shows the optimized geometry for morin deprotonated at 2’-OH [74] calculated at the DFT B3LYP/6-311++G** level of theory. The structure is non planar with a dihedral angle C3-C2-C1’-C2’ equal to 27.6° and it possesses a 1.438 Å hydrogen bond between 3OH and 2’-O^-^ (blue dashed line) in agreement with Musialik et al. proposal. The HOMO of morin shows localized electron density over the C2-C3 double bond (Fig 6B); therefore affecting the chemical reaction rate with singlet oxygen. On the other hand, kaempferol and quercetin neutral optimized geometry are planar (data not shown) and even though their HOMO also show electron density on the C2-C3 double bond, this is delocalized due to resonance. Furthermore, absence of an hydroxyl group in ring B avoids the formation of hydrogen bond with 3OH. The increased acidity besides the enhanced nucleophilicity of enolic bond could explain to the higher chemical reactivity observed for morin.

**Fig 7:**
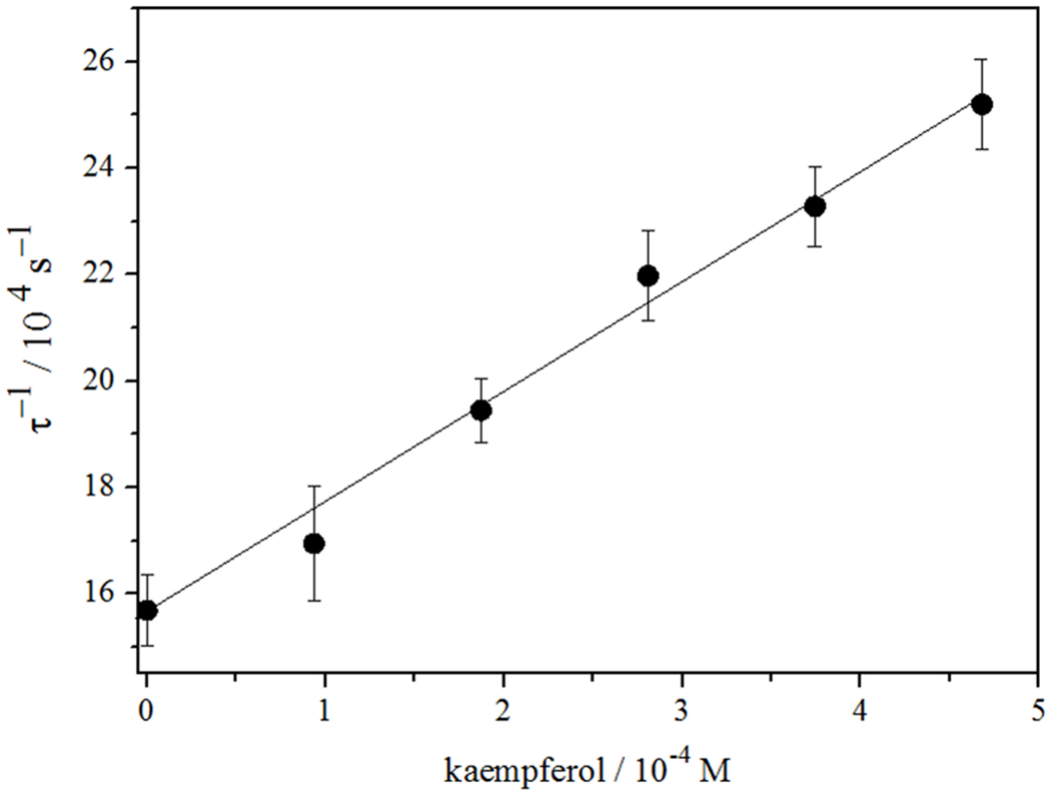
Stern—Volmer type plot for singlet oxygen deactivation by kaempferol in erythrocyte membranes at 25°C.

In this work, the total rate constants (k_T_) for the reaction of singlet oxygen with flavonoids in erythrocyte membranes dispersed in D_2_O (pD 7.4) were obtained by measuring the first-order rate decay (Eq 7) of luminescence in the presence and absence of each flavonoid.

$$\tau^{-1}={\;\tau }_{0}^{-1}+{\;\text{k}}_{\text{T}}\left[\text{FLAV}\right]$$ (7)

where τ^-1^ is singlet oxygen lifetime in presence of flavonoid and τ_0_^−1^ is singlet oxygen lifetime in the absence of FLAV (τ_0_^−1^ = 1/k_d_). Values of k_T_ were calculated from slopes of τ^−1^ vs. [FLAV] plots. Fig 7 shows Stern-Volmer plot for singlet oxygen deactivation by kaempferol in erythrocyte ghost membranes.

The determination of k_T_ was restricted to a narrow range of flavonoid concentration, due to low solubility. All compounds show similar total rate constants being for kaempferol the lower one (2.1x10^7^ M^-1^s^-1^), then quercetin (4.3x10^7^ M^-1^s^-1^) and finally morin being the highest (14.4x10^7^ M^-1^s^-1^). The total rate constants agree to those reported previously [31]. According to literature singlet oxygen deactivation is not influenced by ring A, but the catechol group (ring B) is involved in physical quenching [29–31].

In heterogeneous media (vesicles, biological membranes or cells), the evaluation of the antioxidant capacity is more complex than in homogeneous media, because there are multiple factors involved, such as lipophilicity, antioxidant location, generation, lifetime and diffusion of ROS, among others. The thickness of the lipid bilayer is frequently compared with a polymer film, where degradation processes have been shown to depend on the site of generation of singlet oxygen and its diffusion. Ogilby et al, studied poly(methyl methacrylate) films containing cinnamic acid, and observed dependence of oxygen diffusion coefficients with the extent of cross-linking in polymer [83, 84]. Also, Ogilby et al. have shown that polymer degradation with singlet oxygen depends on the site of generation of the excited specie. Oxygenation is pronounced and extensive when singlet oxygen is generated within the polymeric film, but when it is produced at the polymer surface the reactions are restricted to the surface [85, 86]. In thin superficial layer, the kinetics of local reactions can be diffusion-controlled or not diffusion-controlled depending on heterogeneity of reactants distribution [87].

The deactivation of singlet oxygen by different quenchers in solid organic polymers and in liquid analogs of polymers shows that bimolecular quenching rate constants (kq) for efficient quenchers are smaller than for homogeneous solvents because quenching process in polymers is mainly controlled by solute diffusion to yield the singlet oxygen-quencher encounter pair. The solid media has a leveling effect on the magnitude of quenching rate constants, minimizing the marked differences which can be observed in liquids [88].

In this work, the deactivation of singlet oxygen by quercetin, kaempferol and morin, flavonoids located at the interface of the vesicles and/or erythrocyte membranes, shows no significant difference with those reported in a homogeneous medium. Lipid membranes are less rigid than solid polymer providing less control for diffusion of singlet oxygen-flavonoid encounter pair.

## Concluding Remarks

### Partition and distribution

The order of hydrophobicity of flavonoids studied are: kaempferol ≥ quercetin > morin. The good correlation obtained for the plot of log k´ vs log D (n-octanol/ pH 7.4 buffer) for the studied flavonoids (Fig 3) confirms that k’ as a good hydrophobicity predictor for these systems.

### Relative location in micro-heterogeneous media

Laurdan GP these three compounds promote similar alterations in liposomes and erythrocyte membranes. DPH behavior allows to discard the location of these antioxidants in deeper regions of the lipid bilayer.

### Reactivity of flavonoids with singlet oxygen in microheterogeneous media

The order of reactivity obtained is similar in all micro-heterogeneous and homogeneous studied media (kaempferol < quercetin < morin). No significant changes were seen in the reactivity depending on the phospholipid chains flexibility (POPC) or in the presence of proteins (erythrocyte membranes). Localization and reactivity towards singlet oxygen of quercetin, morin and kaempferol in liposomes (POPC, DPPC and mixtures) and in erythrocyte membranes is highly influenced by the presence of ionized specie. The higher acidity of morin besides the nucleophilicity enhancement of C2-C3 double bond achieved through hydrogen bonding with 2’-O^-^. could explain its higher reactivity.
